# Tissue-Specific Function of *Period3* in Circadian Rhythmicity

**DOI:** 10.1371/journal.pone.0030254

**Published:** 2012-01-11

**Authors:** Julie S. Pendergast, Kevin D. Niswender, Shin Yamazaki

**Affiliations:** 1 Department of Biological Sciences, Vanderbilt University, Nashville, Tennessee, United States of America; 2 Tennessee Valley Healthcare System, Nashville, Tennessee, United States of America; 3 Division of Diabetes, Endocrinology and Metabolism, Department of Medicine, Vanderbilt University School of Medicine, Nashville, Tennessee, United States of America; Nagoya University, Japan

## Abstract

The mammalian circadian system is composed of multiple central and peripheral clocks that are temporally coordinated to synchronize physiology and behavior with environmental cycles. Mammals have three homologs of the circadian *Period* gene (*Per1, 2, 3*). While numerous studies have demonstrated that *Per1* and *Per2* are necessary for molecular timekeeping and light responsiveness in the master circadian clock in the suprachiasmatic nuclei (SCN), the function of *Per3* has been elusive. In the current study, we investigated the role of *Per3* in circadian timekeeping in central and peripheral oscillators by analyzing PER2::LUCIFERASE expression in tissues explanted from C57BL/6J wild-type and *Per3^−/−^* mice. We observed shortening of the periods in some tissues from *Per3^−/−^* mice compared to wild-types. Importantly, the periods were not altered in other tissues, including the SCN, in *Per3^−/−^* mice. We also found that *Per3*-dependent shortening of endogenous periods resulted in advanced phases of those tissues, demonstrating that the *in vitro* phenotype is also present *in vivo*. Our data demonstrate that *Per3* is important for endogenous timekeeping in specific tissues and those tissue-specific changes in endogenous periods result in internal misalignment of circadian clocks in *Per3^−/−^* mice. Taken together, our studies demonstrate that *Per3* is a key player in the mammalian circadian system.

## Introduction

Temporal processes are controlled by circadian clocks, which produce self-sustained oscillations in physiology and behavior with endogenous periods of approximately 24 hours that can be synchronized to environmental cues such as the light-dark cycle and food availability. Circadian clocks are present in the brain and in peripheral tissues and their rhythms are coordinated by a master clock in the suprachiasmatic nuclei (SCN) of the hypothalamus.

The identification of *Period* gene mutants in *Drosophila* was a seminal achievement that fostered the development of the molecular timekeeping model that permeates the circadian field today [1]. The discovery of three homologs of the *Period* gene in mammals (*Per1*, *2*, and *3*) generated excitement that each *Period* gene may have an important function in circadian clocks, which has proven true for *Per1* and *Per2* in rodents. *Per1^−/−^/Per2^−/−^* double mutant mice are arrhythmic, implicating these two *Per* genes as essential components of the SCN molecular timekeeping machinery [2]. In addition, the expression of both *Per1* and *Per2* mRNAs is acutely induced in the SCN by exposure to light pulses and *Per1^−/−^* and *Per2^−/−^* mice have distinct patterns of altered light responsiveness [3], [4], [5]. In contrast, *Per3^−/−^* mice have no overt circadian behavioral phenotypes [2], [3], [6], [7]. These early studies led to the conceptualization that *Per3* does not play an important role in the mammalian circadian system.

Recently, renewed interest in *Per3* has centered on its non-circadian functions. Studies of humans have demonstrated that differences in sleep homeostasis are associated with the PER3 variable number tandem repeat (VNTR) polymorphism [8], [9], [10]. Recent studies in mice also reported roles for *Per3* in regulating sleep/wake timing, sleep homeostasis, and retinal physiology [11], [12]. *Per3* may also be important in regulating metabolism and body composition. *Per3^−/−^* mice gain more weight than wild-type controls when fed high-fat diet [13] and PER3 is an inhibitor of adipocyte cell fate, which results in greater adiposity in *Per3^−/−^* mice compared to wild-types [14].

In addition to these non-circadian functions of *Per3*, we recently reported that *Per3* regulates the period and phase of circadian rhythms in pituitary and lung [7]. These findings suggest that the early studies of *Per3* function in the circadian system, which focused on SCN-dependent behavior and light responsiveness, may have inadvertently dismissed *Per3* as an important player in the mammalian circadian system. In this study, we further investigated the role of *Per3* in circadian timekeeping in central and peripheral clocks.

## Materials and Methods

### Animals

We obtained *mPer3^−/−^* mice [6] (provided by Dr. David Weaver, University of MA, congenic with the 129/sv genetic background) and backcrossed them with wild-type C57BL/6J mice (Jackson Laboratory, Bar Harbor, ME) for at least 15 generations [7] (C57BL/6J *Per3^−/−^* mice are available from The Jackson Laboratory, stock #10493). To generate luciferase reporter mice, C57BL/6J *mPer3^+/−^* mice were crossed with C57BL/6J heterozygous PER2::LUCIFERASE mice [15] (PER2::LUC mice were backcrossed to wild-type C57BL/6J mice from The Jackson Laboratory for at least 16 generations) to generate mice that were heterozygous for both the *Period3* gene and for the PER2::LUC knock-in gene. *Period3* heterozygous (without the PER2::LUC gene) mice were then crossed with *Period*3 heterozygous mice with the PER2::LUC gene to generate wild-type and homozygous mutant *Per3* mice that were heterozygous for PER2::LUC that were used for experiments. Genotyping for the *Per3* gene was performed as previously described [6] and the presence of the PER2::LUC fusion protein was determined by measuring light emission from a fresh tail piece using a luminometer. The mice were bred and group-housed in the Vanderbilt University animal facility in a 12h-light/12h-dark cycle (12L∶12D; light intensity ∼350 lux) and provided food and water *ad libitum*. Male mice were used for all experiments. The mean (± SD) ages of the mice at the time of culture were: wild-types: 113±44 days; *Per3^−/−^* mice: 108±34 days. All experiments were conducted in accordance with the guidelines of the Institutional Animal Care and Use Committee at Vanderbilt University.

### Luminescence recording

Cultures were prepared within 1.5 hrs before lights off, as previously described, except that CellGro (cat. no. 90-013PB plus L-glutamine) recording medium was used [16]. Since rapid dissection of tissues is critical for preventing resetting of circadian phase, and we sought to analyze multiple tissues, we could not simultaneously collect all tissues from a single mouse. White adipose tissue (from above the adrenal gland), adrenals, esophagus, kidney, liver, lung, spleen, and thymus were collected from the same mouse, while olfactory bulbs, aorta, colon, gonadal white adipose tissue (surrounding the gonads), liver, pituitary, SCN, arcuate complex (containing the arcuate nucleus of the hypothalamus and ependymal cell layer as described previously [17]), pituitary, and SCN were collected from a different mouse. We collected the olfactory bulbs on numerous occasions (and from many different regions of the bulbs-rostral to caudal and whole vs. core or shell), but we were not able to reliably obtain a rhythm that could be analyzed. Bioluminescence was monitored in real-time with the LumiCycle, and photon counts were integrated over 10-minute intervals. LumiCycle software (Actimetrics Inc., Wilmette, IL) was used to subtract the 24-hour moving average from the raw luminescence data and to smooth the data by 0.5-hour adjacent averaging. To determine period and phase, the detrended and smoothed data were exported to ClockLab (Actimetrics Inc., Wilmette, IL). The period was determined by fitting a regression line to the acrophase of at least 3 days of the PER2::LUC rhythm and the phase was determined from the peak of PER2::LUC expression during the interval between 12 h and 36 h in culture.

### Statistical analysis

Statistical analysis was performed using SigmaStat (Systat Software, Inc., San Jose, CA). Independent *t* tests (two-tailed) were used to compare two groups. The liver period data were not normally distributed [as determined by the Kolmogorov-Smirnov test (with Lilliefors' correction)], so the Mann-Whitney Rank Sum test was used for comparison. Significance was ascribed at *p*<0.05.

## Results

**Figure 1:**
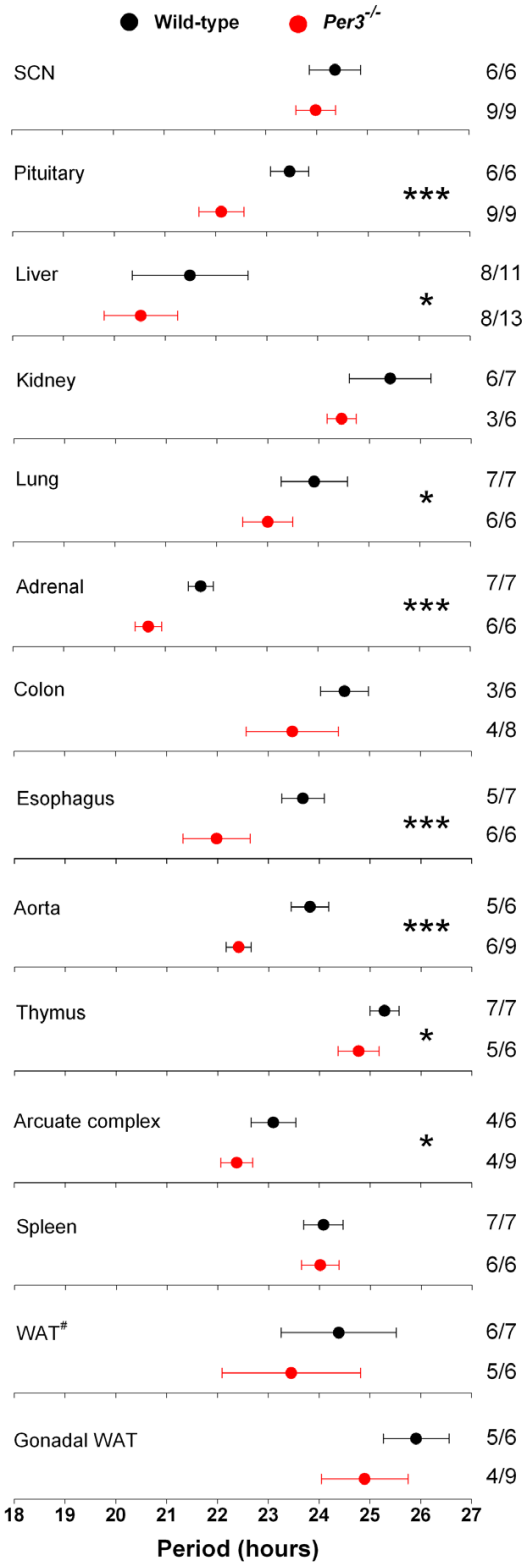
Tissue-specific alterations of circadian periods in *Per3^−/−^* mice. Bioluminescence was recorded from tissue explants prepared from male wild-type (black circles) and *Per3^−/−^* (red circles) mice maintained in 12L∶12D. The mean (± SD) periods were determined by fitting regression lines to the acrophases of the PER2::LUC rhythms. The sample size is shown (number of rhythmic tissues/number of tissues tested). WAT^#^: white adipose tissue surrounding the adrenal gland. *p<0.05; **p<0.01; ***p<0.001.

To examine the role of *Per3* in the endogenous timekeeping mechanisms in central and peripheral tissues, we assessed PER2::LUC expression in cultured tissues explanted from C57BL/6J wild-type and *Per3^−/−^* mice. We found that the periods of PER2::LUC expression in pituitary, liver, lung, adrenals, esophagus, aorta, thymus, and arcuate complex were shorter in *Per3^−/−^* mice compared to wild-types (Figure 1, Table 1). In contrast, the periods of PER2::LUC expression in SCN, kidney, colon, spleen, white adipose tissue (surrounding the adrenal gland), and gonadal white adipose tissue were not altered by the loss of functional PER3 (Figure 1, Table 1).

**Table 1:** Tissue-specific differences in periods and phases between wild-type and *Per3^−/−^* mice.

Tissue	Period difference (h)	Phase difference (h)
SCN	NS	NS
Pituitary	1.34	5.01
Liver	0.97	4.46
Kidney	NS	NS
Lung	0.92	1.67
Adrenal	1.03	NS
Colon	NS	4.01
Esophagus	1.70	3.05
Aorta	1.40	1.88
Thymus	0.51	NS
Arcuate complex	0.72	NS
Spleen	NS	NS
WAT	NS	NS
Gonadal WAT	NS	1.72

The differences in the periods (h) and phases (h) were calculated by subtracting the mean *Per3^−/−^* value from the mean wild-type value for each tissue (the periods and phases of the *Per3^−/−^* tissues were always shorter or advanced, respectively, compared to wild-types). NS: No statistically significant difference detected.

**Figure 2:**
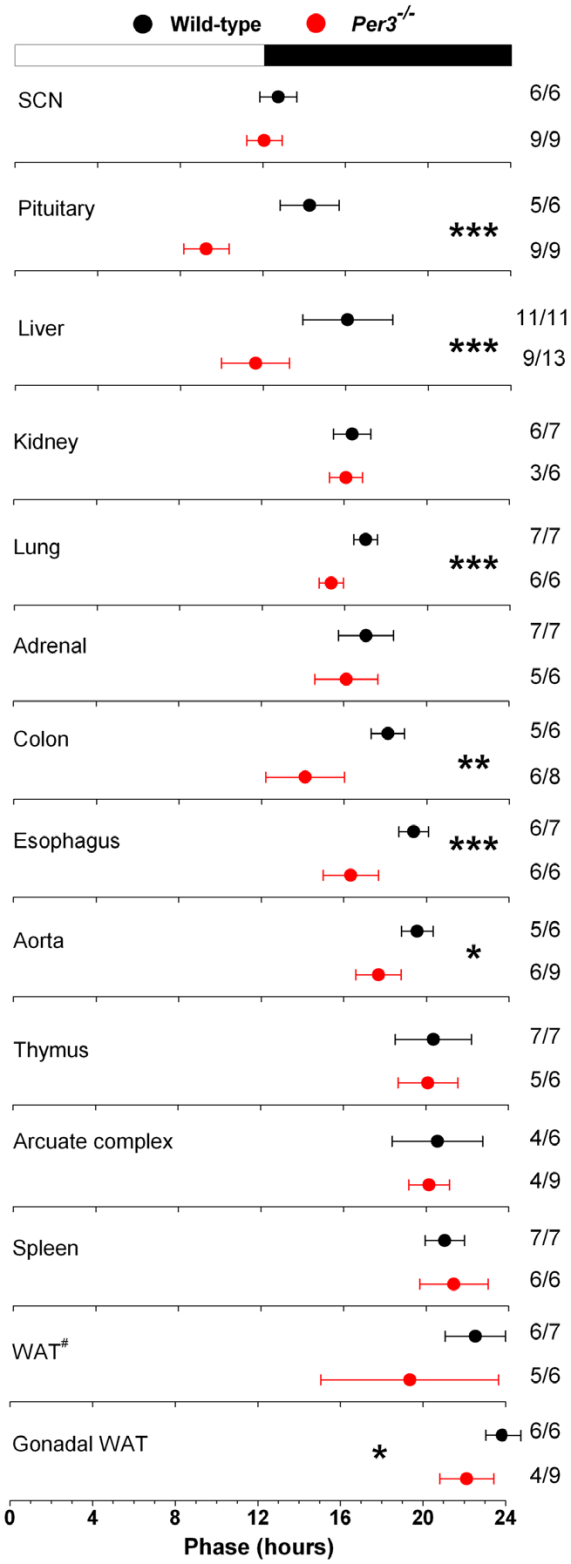
Altered circadian organization in *Per3^−/−^* mice. Bioluminescence was recorded from tissue explants prepared from male wild-type (black circles) and *Per3^−/−^* (red circles) mice maintained in 12L∶12D. The mean phases (± SD) were determined from the peaks of PER2::LUC expression during the interval between 12 h and 36 h in culture and were plotted relative to the time of last lights-on, where 0 h is lights on and 12 h is lights off (black and white bar at top). The sample size is shown (number of rhythmic tissues/number of tissues tested). WAT^#^: white adipose tissue surrounding the adrenal gland. *p<0.05; **p<0.01; ***p<0.001.

To assess the effect of loss of functional PER3 on circadian organization, we analyzed the phases of PER2::LUC expression in tissues explanted from wild-type and *Per3^−/−^* mice (Figure 2, Table 1). We found that circadian organization was altered in *Per3^−/−^* mice such that the phases of PER2::LUC expression were advanced in pituitary, liver, lung, colon, esophagus, aorta, and gonadal white adipose tissue compared to wild-type mice. The phases of SCN, kidney, adrenals, thymus, arcuate complex, spleen, and white adipose tissue were not altered in *Per3^−/−^* mice compared to wild-types.

**Figure 3:**
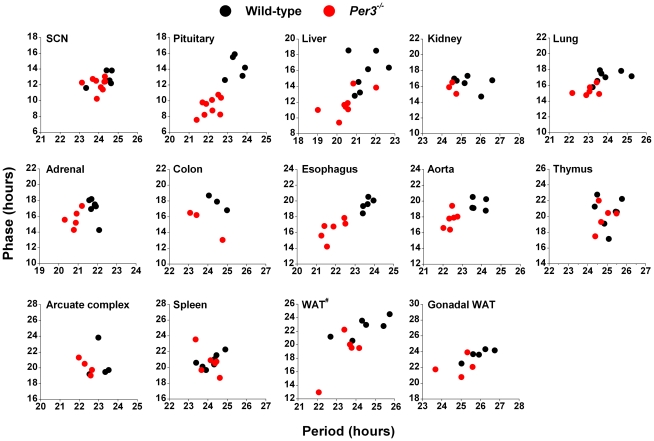
Tissue-specific relationships between phase and period in wild-type and *Per3^−/−^* mice. The phase of each sample was plotted relative to its endogenous period of PER2::LUC expression. All samples for which both phase and period could be analyzed are shown. Wild-type (black circles) and *Per3^−/−^* (red circles) samples are plotted on the same graph for each tissue. WAT^#^: white adipose tissue surrounding the adrenal gland.

To determine if the altered endogenous periods caused by loss of functional PER3 were reflected in circadian phases, we plotted the phase of each sample relative to its endogenous period (Figure 3). We found that in tissues where the endogenous period was affected by the loss of functional PER3 (pituitary, liver, lung, esophagus, aorta), the short endogenous periods resulted in advanced phases. In the SCN, kidney, thymus, arcuate complex, and spleen, endogenous periods were not reflected in the phases of PER2::LUC expression.

## Discussion

Identification of the function of *Per3* in the mammalian circadian system has been elusive and nearly dismissed, until recently. Studies of humans were the first to identify a putative physiological function of *Per3*, in the regulation sleep [9], [10]. Animals studies were unsuccessful in identifying functional roles for *Per3* in circadian behavior, light responsiveness, and sleep [6], [7], [18]. However, these studies largely focused on behavior and physiology related to the function of the master circadian clock in the SCN. Recently we found that the periods and phases of the pituitary and lung, but not the SCN, were altered in *Per3^−/−^* mice, suggesting that we may have been searching for the functional role of *Per3* in the wrong oscillator [7]. The mammalian circadian system is composed of numerous oscillators in the brain and periphery and in the current study we sought to determine the role of *Per3^−/−^* in extra-SCN oscillators.

In the current study, we confirmed that *Per3* does not function in period determination in the SCN. However, we found that the periods of the circadian rhythms in numerous extra-SCN tissues from *Per3^−/−^* mice were altered compared to wild-type mice. Our approach measured the endogenous timekeeping mechanism in tissue explants in culture with no influence from the *in vivo* environment or the SCN. The fact that we observed period shortening in tissues in these experimental conditions suggests that *Per3* is important for timekeeping and period determination in specific extra-SCN oscillators. The function of *Per3* was tissue-specific such that the loss of functional PER3 had no effect on circadian period in about half of the tissues we analyzed. Whether the tissue-specific nature of *Per3* function is related to the physiological outputs of the tissues could be an interesting focus of future studies.

Since the phase of each tissue is an integration of its endogenous period with *in vivo* inputs, we next determined whether the alterations in the endogenous timekeeping mechanisms in tissues from *Per3^−/−^* mice were reflected in the phases of their circadian rhythms. We found that *Per3*-dependent shortening of endogenous periods resulted in the advanced phase of those tissues, demonstrating that the *in vitro* phenotype is also present *in vivo*.

The periods and phases of the SCN and some extra-SCN oscillators were not altered, while the phases of many other tissues were advanced, resulting in internal misalignment of circadian clocks in *Per3^−/−^* mice relative to wild-types. Central and peripheral clocks acquire a specific phase relationship with each other that is believed to optimally coordinate behavior and physiology with environmental cycles [19]. Distortion of the phase relationship between these clocks by jet-lag and shift work is associated with poor health, including obesity, increased cancer risk, depression, sleep disturbances, and premature death [20]. We predict that careful analyses of multiple physiological parameters in *Per3^−/−^* mice will reveal abnormal phenotypes that may be related to the misalignment of the phases of their oscillators. Consistent with this prediction, aberrant metabolic and sleep phenotypes have already been reported in *Per3^−/−^* mice [11], [13], [14].

In conclusion, we found that *Per3* is important for endogenous timekeeping in specific tissues. Furthermore, tissue-specific changes in endogenous periods result in altered circadian organization in *Per3^−/−^* mice. Future studies examining the physiological ramifications of internal misalignment in *Per3^−/−^* mice will further elucidate the role of *Per3* in the circadian system. Finally, our studies demonstrate that *Per3* is a key player in the mammalian circadian system.

## References

[1] Konopka RJ, Benzer S (1971). Clock mutants of Drosophila melanogaster.. Proc Natl Acad Sci U S A.

[2] Bae K, Jin X, Maywood ES, Hastings MH, Reppert SM (2001). Differential functions of mPer1, mPer2, and mPer3 in the SCN circadian clock.. Neuron.

[3] Pendergast JS, Friday RC, Yamazaki S (2010). Photic Entrainment of Period Mutant Mice is Predicted from Their Phase Response Curves.. J Neurosci.

[4] Albrecht U, Zheng B, Larkin D, Sun ZS, Lee CC (2001). MPer1 and mper2 are essential for normal resetting of the circadian clock.. J Biol Rhythms.

[5] Spoelstra K, Albrecht U, van der Horst GT, Brauer V, Daan S (2004). Phase responses to light pulses in mice lacking functional per or cry genes.. J Biol Rhythms.

[6] Shearman LP, Jin X, Lee C, Reppert SM, Weaver DR (2000). Targeted disruption of the mPer3 gene: subtle effects on circadian clock function.. Mol Cell Biol.

[7] Pendergast JS, Friday RC, Yamazaki S (2010). Distinct functions of Period2 and Period3 in the mouse circadian system revealed by in vitro analysis.. PLoS One.

[8] Dijk DJ, Archer SN (2010). PERIOD3, circadian phenotypes, and sleep homeostasis.. Sleep Med Rev.

[9] Goel N, Banks S, Mignot E, Dinges DF (2009). PER3 polymorphism predicts cumulative sleep homeostatic but not neurobehavioral changes to chronic partial sleep deprivation.. PLoS One.

[10] Viola AU, Archer SN, James LM, Groeger JA, Lo JC (2007). PER3 polymorphism predicts sleep structure and waking performance.. Curr Biol.

[11] Hasan S, van der Veen DR, Winsky-Sommerer R, Dijk DJ, Archer SN (2011). Altered sleep and behavioral activity phenotypes in PER3-deficient mice.. Am J Physiol Regul Integr Comp Physiol.

[12] van der Veen DR, Archer SN (2010). Light-dependent behavioral phenotypes in PER3-deficient mice.. J Biol Rhythms.

[13] Dallmann R, Weaver DR (2010). Altered body mass regulation in male mPeriod mutant mice on high-fat diet.. Chronobiol Int.

[14] Costa MJ, So AY, Kaasik K, Krueger KC, Pillsbury ML (2011). Circadian rhythm gene period 3 is an inhibitor of the adipocyte cell fate.. J Biol Chem.

[15] Yoo SH, Yamazaki S, Lowrey PL, Shimomura K, Ko CH (2004). PERIOD2::LUCIFERASE real-time reporting of circadian dynamics reveals persistent circadian oscillations in mouse peripheral tissues.. Proc Natl Acad Sci U S A.

[16] Yamazaki S, Takahashi JS (2005). Real-time luminescence reporting of circadian gene expression in mammals.. Methods Enzymol.

[17] Guilding C, Hughes AT, Brown TM, Namvar S, Piggins HD (2009). A riot of rhythms: neuronal and glial circadian oscillators in the mediobasal hypothalamus.. Mol Brain.

[18] Shiromani PJ, Xu M, Winston EM, Shiromani SN, Gerashchenko D (2004). Sleep rhythmicity and homeostasis in mice with targeted disruption of mPeriod genes.. Am J Physiol Regul Integr Comp Physiol.

[19] Yamazaki S, Numano R, Abe M, Hida A, Takahashi R (2000). Resetting central and peripheral circadian oscillators in transgenic rats.. Science.

[20] Arendt J (2010). Shift work: coping with the biological clock.. Occup Med (Lond).

